# Meniscal sutures: biomechanical study of “mulberry” and horizontal loop techniques

**DOI:** 10.1007/s10195-011-0162-y

**Published:** 2011-12-22

**Authors:** Fabiano Fantasia, Gabriele Potalivo, Giacomo Placella, Luigi Fantasia, Giuliano Cerulli

**Affiliations:** 1 Department of Orthopaedics, Nuova Clinica San Francesco, Foggia, Italy; 2 Department of Orthopaedics and Traumatology, S. Maria della Misericordia Hospital, Orthopaedic and Traumatology Recidency Program, University of Perugia, 06156 S Andrea delle Fratte, PG Italy

**Keywords:** Arthroscopy, Meniscus, Sutures, Loop, Mulberry

## Abstract

**Background:**

This in vitro biomechanical study tested the pullout strength of meniscal repair in human menisci using two different biodegradable suture techniques: the “mulberry” and the horizontal loop.

**Materials and methods:**

Fifty-five human menisci were used, to which a longitudinal tear of 1.5 cm was applied. If the thread broke or the knot was pulled inside the suture, as happened with the mulberry technique, the repair was considered a failure. Furthermore, we evaluated possible lesions of the meniscus due to changes the structural properties caused by the suture, leading to the loss of elastic return.

**Results:**

The results showed there was a statistically significant difference between the two suture techniques used and the unsutured menisci. Furthermore, five menisci with vertical sutures were analyzed for which the breakup loads were superior to the breakup loads of the mulberry suture and the horizontal loop suture. Nevertheless, the load strengths with respect to elastic return were similar to those of the mulberry and the horizontal loop techniques. Finally, in five menisci, we analyzed the suture–healthy meniscus interface, and found breakup values similar to those of the unsutured meniscus.

**Conclusions:**

Our results show the need to perform meniscal sutures and the futility of sutures that are intended to withstand elevated loads such as traction strengths of >30 N, as these produce irreparable secondary lesions that alter the histological structure of the meniscus and prevent healing.

## Introduction

Normal menisci protect the joint cartilage by distributing loads over a broad and congruent area in order to guarantee stability and correct movements of the joint surfaces, lubricating and nourishing them [1–4]; moreover they provide propioceptive information [5].

The decision to repair a meniscus is preferable, but it is necessary to carefully evaluate several aspects, such as the type of injury, the site, the quality of the tissue, the patient’s age, and the blood supply. Analysis of these factors allows us to foresee the recovery potential of the injury and to exclude recurrences.

Arnoczky and Warren’s [6] studies have given us an insight into the meniscal microvascular distribution. Meniscal repair is directly correlated with the degree of vascularization and the ability to generate an inflammatory-type healing response, which is a physiopathological mechanism that depends strongly on the patient’s age. Radial injuries that extend as far as the vascularized synovial need at least ten weeks to heal, while the scar needs several months to become fibrocartilage [2, 7]. This situation has led to a debate over whether it is advisable to use reabsorbable devices, especially in cases where an accelerated postoperative rehabilitation protocol has been chosen [8].

Various techniques are available for meniscal repair: “in–out,” “out–in,” and “all inside.”

Some reports in the literature indicate tensile breakage strengths of 115 N for vertical loop sutures, 75 N for horizontal loop sutures, and only 38 N for biodegradable arrows [9–11]. Other authors [12] report different data: 63.7 N for the single vertical loop stitch and 29.3 N for the horizontal loop suture. Considering the lack of accord among the available data and the greater reliability of sutures compared to arrows, the aim of this study was to compare the breakage tensile strengths of two simple and effective arthroscopic suture techniques: the “mulberry” method, as first described by Cooper, Arnoczky and Warren in 1990 [13], and the horizontal loop technique, as described by Johnson in 1995 [14] (both can be performed with either the in–out or the out–in technique). Furthermore, the study took into consideration possible methods of making this type of suture more reliable.

## Materials and methods

Fifty-five human menisci with intact capsules and no evidence of previous injury or degenerative changes were examined.

**Fig. 1:**
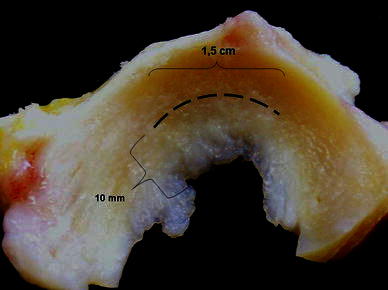
A 1.5 cm longitudinal incision was made with a number 21 scalpel, leaving 10 mm of tissue from the internal meniscal border

The tests were performed immediately after harvesting. A 1.5 cm longitudinal incision was made in the red–white area with a number 21 scalpel, leaving the tissue surrounding the tear as undamaged as possible (Fig. 1).

**Fig. 2:**
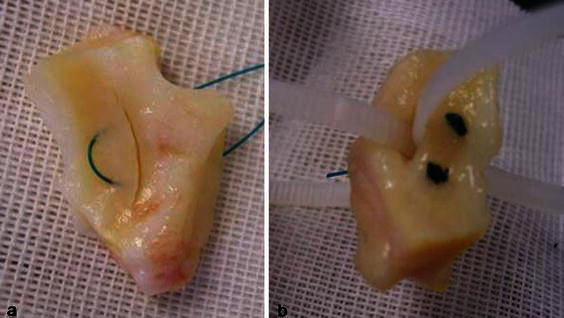
The meniscal lesion was repaired with a monofilament polyglyconate synthetic absorbable suture (MAXON 2-0) and a 21G needle in each technique. **a** The horizontal loop was made by inserting the suture into the peripheral half of the meniscal tissue with an initial out–in step and then an in–out step. **b** The mulberry was performed with one double knot and two half hitches. Two plastic tapes were inserted into the tear

**Fig. 3:**
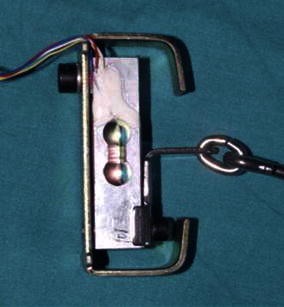
A perpendicular force was applied to the collagen fibers surrounding the meniscus circumferentially using a custom-designed mechanical system together with a load cell equipped with a digital system to read the data

The meniscal lesion was repaired with a monofilament polyglyconate synthetic absorbable suture (MAXON 2-0) and a 21G needle in each technique (Fig. 2a). The mulberry was performed with one double knot and two single ones. By placing two plastic tape loops inside the tear (Fig. 2b), we were able to apply a perpendicular force to the collagen fibers surrounding the meniscus circumferentially [15] by means of a custom-designed mechanical system together with a load cell equipped with a digital system to read the data (Fig. 3).

**Fig. 4:**
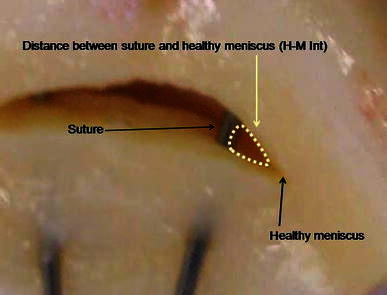
The distance between the suture and healthy meniscus was assessed, a distance that we call the “healthy meniscus–suture interface” (H–M int). This area is a weak point like an unsutured lesion

**Fig. 5:**
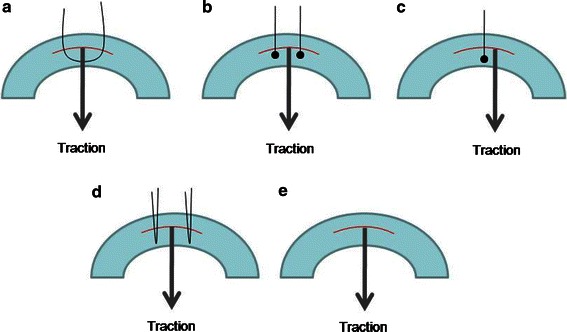
We analyzed **a** 15 meniscal lesions repaired using a horizontal loop suture with one thread, **b** 15 lesions repaired with a mulberry suture with two threads, **c** five meniscal tears sutured with one thread (to evaluate the healthy meniscus–suture interface; see main text), **d** five meniscal tears with a double vertical suture, and **e** five unsutured meniscal lesions. The *gray arrow* indicates the point of traction

Fifteen meniscal lesions repaired with mulberry sutures with two seperate threads and 15 lesions repaired with horizontal loop sutures were evaluated after applying cyclic and increasing loading. Furthermore, we analyzed 15 unsutured meniscal lesions, five meniscal tears sutured with a single thread in which the distance between the suture and healthy meniscus was assessed—a distance that we call the “healthy meniscus–suture interface” (H–M int), (Fig. 4), and five meniscal tears with a double vertical suture (Fig. 5).

A broken thread was considered a failed suture; moreover, when the knot doubled up in the meniscus in the mulberry suture, this was also considered a failure. We used an unsutured meniscus as a control, and we analyzed the tensile behavior of the zone interposed between the first suture and healthy meniscus (the healthy meniscus–suture interface, for both mulberry and for horizontal loop sutures) by determining the force needed to produced a significant lesion or meniscal rupture. The results of such tests are important for evaluating the best zone in which to place a suture.

The aim of the study was to establish which suture was the most resistant to traction. Furthermore, we measured the maximum load that can be applied while preserving the structural properties of the meniscus without the suture causing secondary lesions (an event defined as “elastic return”).

Finally, we evaluated the best suture position and defined the concept of a “healthy meniscus–suture interface.”

## Results

The results obtained, which are summarized in Table 1, do not show any statistically significant differences between the mulberry and the horizontal loop sutures for the break load (94.09 ± 6.77 N vs. 105.33 ± 5.71 N, *p* > 0.05) and the elastic return (26.22 ± 2.03 vs. 25.83 ± 2.04, *p* > 0.05), but there were statistically significant differences in these parameters between the two suture methods used and the unsutured meniscus. The break loads of the mulberry technique and the unsutured meniscus were 94.09 ± 6.77 N and 38.85 ± 0.93 N, respectively (*p* < 0.0001); the break loads of the horizontal loop suture and the unsutured meniscus were 105.33 ± 5.71 N and 38.85 ± 0.93 N, respectively (*p* < 0.0001).

**Table 1:** Rupture load and elastic return values (expressed in newtons) for the menisci sutured using the mulberry technique, the horizontal loop technique, or the vertical loop technique, as well as the injured and unsutured menisci and the healthy meniscus–suture interface (H–M Int)

	Mean traction load				
	Mulberry	Horizontal loop	No suture	Vertical loop	H–M interface
Elastic return	26.22 ± 2.03	25.83 ± 2.04	18.99 ± 0.16	25.9 ± 2.08	19.45 ± 0.22
Rupture	94.09 ± 6.77	105.33 ± 5.71	38.85 ± 0.93	123.8 ± 3.08	40.86 ± 2.11

Comparison of behavior	*p* value
Mulberry versus horizontal loop: rupture	0.2144
Mulberry versus horizontal loop: elastic return	0.8924
Mulberry versus no suture: rupture	0.0001
Horizontal loop versus no suture: rupture	0.0001
Horizontal loop versus no suture: elastic return	0.0023
Mulberry versus no suture: elastic return	0.0013
No suture versus H–M interface: elastic return	0.1508
No suture versus H–M interface: rupture	0.3294
Mulberry versus vertical loop: elastic return	0.9314
Vertical loop versus horizontal loop: elastic return	0.9866
Mulberry versus vertical loop: rupture	0.024
Horizontal loop versus vertical loop: rupture	0.0871

When the elastic return is considered, the traction loads applied to the mulberry suture and the unsutured meniscus were found to be statistically significantly different (26.22 ± 2.03 vs. 18.99 ± 0.16, *p* < 0.005), as were the traction loads applied to the horizontal loop suture and the unsutured meniscus (25.83 ± 2.04 vs. 18.99 ± 0.16, *p* < 0.005). Five menisci with vertical sutures were also analyzed, and their break loads were found to be 123.8 ± 3.08 N, similar to data reported in literature [10–14], and greater than the break loads of the menisci sutured using the mulberry (statistically significant difference: *p* < 0.05) and the horizontal loop (no statistically significant difference: *p* = 0.08) techniques. However, the load strength for elastic return of the menisci sutured using the vertical loop method was 25.9 ± 2.08 N, similar to those of the mulberry and the horizontal loop techniques (no statistically significant difference: *p* > 0.05). Finally, the healthy meniscus–suture interface was evaluated in five menisci, which presented a breakage value of 40.86 ± 2.11 N and an elastic return of 19.45 ± 0.22 N, neither of which were significantly different from the corresponding values for the unsutured meniscus (*p* > 0.05 for both assessments).

## Discussion

Much has changed in the last few years with regards to meniscal repair, due to our enhanced knowledge of the role of menisci as well as improvements in surgical techniques.

In the literature, there is much debate over whether to use the horizontal or vertical loop suture techniques. Some authors [16] have observed no differences in tension between vertical and horizontal sutures, whereas others believe that horizontal sutures are less effective (less than 25% of the rupture strength of vertical sutures) [17]. Numerous studies have reported that vertical sutures for meniscal repair provide superior load to failure compared to horizontal sutures [10, 15, 18–20]. This could be because the vertical loop captures a greater proportion of the semicircularly oriented meniscal collagen fibers.

Song et al. [10] obtained a superior load to failure with vertical sutures (113.9 ± 14.6 N) compared to horizontal sutures (75.1 ± 18.4 N) using a 0-PDS suture in a porcine model.

Post et al. [21] used a young porcine medial and a lateral meniscus model and reported that the load to failure using vertical sutures (146.3 ± 17.1 N) and (115.9 ± 28.5 N), respectively, was superior to that obtained with horizontal sutures (73.81 ± 31.3 N) and (66.1 ± 28.7 N) when 1-PDS and 0-PDS were used, respectively. Additionally, they reported comparable load to failure results for repairs using horizontal sutures regardless of whether 2–0 Ethibond (59.7 ± 20.4 N), 0-PDS (66.1 ± 28.7 N), or 1-PDS (73.81 ± 31.3 N) were used as suture materials. The selected suture material made a greater contribution to the fixation when vertical sutures were used, with 1-PDS sutures (146.3 ± 17.1 N) showing superior load to failure compared to 0-PDS sutures (115.9 ± 28.5 N).

Kocabey [18] reported that the mean loads to failure of oblique (171.9 ± 25.9 N) and vertical loop (145.9 ± 32.3 N) sutures were not statistically different. The 18% difference in favor of the oblique suture is probably due to the fact that a greater meniscal area is sutured, together with a greater percentage of horizontal fibers.

Another debatable point concerns the use of absorbable or nonabsorbable thread; the first is more elastic and risks being absorbed before the injury has healed, while the second is more rigid and offers greater stability, allowing early and rapid rehabilitation [20], although it remains as a permanent foreign body in the suture site, and may therefore cause secondary lesions later on.

Noting Post’s studies [21] and the implications of using nonabsorbable thread, we chose to use MAXON 2-0, (monofilament polyglyconate synthetic absorbable suture) for all techniques. Size is an important factor in mini-invasiveness, as the use of a finer thread allowed us to use 21G needles, which are less traumatic than the needles used previously (18G), leading to high values of resistance to traction. These considerations mainly apply to the mulberry technique, where the 18G needle made a passage big enough for a knot to pass through, ultimately resulting in the failure of the suture. The 21G needle allowed us to reduce the size of the knot (one double and two single knots) in order to avoid creating a foreign body within the joint, which could be traumatic for the cartilage.

**Fig. 6:**
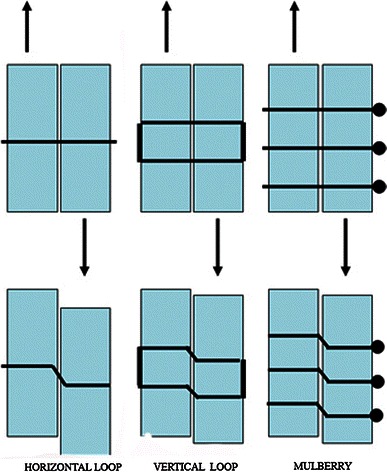
According to Zantop’s theory, the circumferential forces are distributed better by horizontal loop sutures, whereas our data show that mulberry sutures are better

It is therefore obvious how the vertical loop suture is more resistant to traction (distraction) than other types of suture; it should also be noted that the meniscus in the knee, above all, undergoes shear forces. Zantop et al. [21] have assessed the circumferential stress to the meniscal injury while comparing vertical and the horizontal sutures. The horizontal suture presented an elongation of 2.8 ± 1.1 mm, while the elongation was 4.6 ± 2.0 mm for the vertical suture, which proved to be more stable when undergoing this kind of loading. The structure of the mulberry suture offers biomechanical features similar to those of the horizontal loop, guaranteeing a greater resistance to shear force compared to the vertical loop, with the advantage that—as it uses separate threads—one broken thread does not completely jeopardize the success of the suture. Assessments will be performed in the future (Fig. 6).

**Fig. 7:**
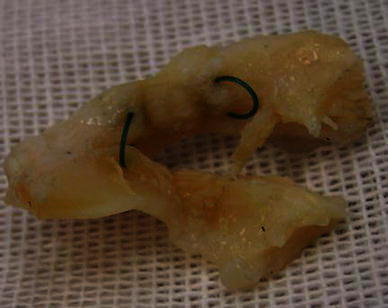
The photo shows the complete loss of meniscal structure due to maximal traction load, despite the fact that the suture did not yield

Nonetheless, in our experiments, we observed that the suture irreparably damaged the meniscus before it yielded. Indeed, what needs to be considered is not so much the suture’s break point but the precise moment at which the meniscus undergoes irreparable damage while being subjected to traction force, which relentlessly alters the microarchitecture and three-dimensional structure, thus completely eliminating its intrinsic elasticity. The suture’s break load therefore loses its meaning if the meniscus is irreparably damaged. The suture’s purpose is therefore to draw together the lesion’s strips and to guarantee an anatomical and lasting fixation that ensures the time needed to achieve recovery is received (Fig. 7). Regarding the elastic return, the differences between the traction loads applied to the mulberry suture and the unsutured meniscus (26.22 ± 2.03 vs. 18.99 ± 0.16, *p* < 0.005) and the horizontal loop suture and the unsutured meniscus (25.83 ± 2.04, *p* < 0.005) were statistically significant.

Finally, in five menisci, the area between the suture and the extremity of the lesion, which we defined as the healthy meniscus–suture interface, was evaluated and presented a rupture value of 40.86 ± 2.11 N and an elastic return of 19.45 ± 0.22 N, which were not statistically significantly different from the values for unsutured menisci (*p* > 0.05 for both measurements). These further assessments led us to consider the healthy meniscus–suture interface to be similar to an unsutured lesion—a weak point that can be corrected by drawing the suture as close as possible to the healthy meniscus. We believe that it is necessary to not only perform a meniscal repair but also pay much attention to where the suture is applied, thereby identifying the weakness area in between the first suture and non-damaged meniscus.

Respecting anatomical and biomechanical principles, our experiments have highlighted the fundamental features and requisites needed to achieve a meniscal suture that allows the injured tissue to heal and the lost structural integrity to recover.

We can now claim that the first factor to consider is the anatomical position at which to perform the suture. In fact, it is necessary to try and draw the suture as close as possible to the healthy meniscus, reducing the space defined as the healthy meniscus–suture interface, as it is an out-and-out weak point when applying the load force. Considering the loss of structural and elastic properties of the meniscus in the presence of traction loads lower than those needed for breakage, and the risk of secondary lesions due to certain load levels, it is understandable why it is necessary and advantageous to draw together only the edges of the meniscal lesion, respecting the physiopathological processes at the root of the healing process. The biomechanical principle according to us is not that of a “quantitative” suture which can withstand high traction loads, but rather a “qualitative” one which is placed correctly and has a certain level of manageability that can grant the necessary healing time. According to these concepts, we do not believe in accelerated rehabilitation, as this could invalidate, complicate, and slow down the healing mechanisms that the meniscus implements, accelerating degenerative processes and possibly causing secondary lesions due to the suture.

As far as the technique is concerned, it would be advisable to choose a slowly absorbable suture capable of offering meniscal stability and withstanding hydrolysis to a sufficient degree, so that it does not yield before the slow healing and recovery process has occurred. It is preferable to use 21G needles of a reduced size, bearing in mind both the mini-invasive approach and the effort that should be expended to reduce the knots, which are potentially harmful to the intra-articular knee structures.

To date, our studies and the literature have not unambiguously identified a particular suture that will withstand both the vertical and the circumferential traction forces which normally occur within the knee, but we feel that the characteristics we have suggested and tested scientifically could provide the foundation for a successful technique.

A simple and mini-invasive technique aimed at drawing together the borders of the tear in an anatomic fashion, reducing the healthy suture–meniscus interface, and respecting the time needed for healing to set in, as well as the use of biodegradable thread and the performance of a biomechanical analysis of the forces within the knee that are unloaded to the meniscus are the basic concepts of meniscal suture. In conclusion, the mulberry-type suture fits well with the abovementioned criteria, which is why we prefer it to the other techniques.

In future studies, we will assess the follow-up in patients with mulberry sutures, evaluate the traction loads both perpendicular and circumferential to the tear in different types of sutures, including biodegradable arrows, and perform a microscopic analysis in order to better understand the three-dimensional histologic structure that can be altered by the suture itself.
